# Towards blockchain based federated learning in categorizing healthcare monitoring devices on artificial intelligence of medical things investigative framework

**DOI:** 10.1186/s12880-024-01279-4

**Published:** 2024-05-10

**Authors:** Syed Thouheed Ahmed, T. R. Mahesh, E. Srividhya, V. Vinoth Kumar, Surbhi Bhatia Khan, Abdullah Albuali, Ahlam Almusharraf

**Affiliations:** 1 Department of Electrical Engineering, Indian Institute of Technology, Hyderabad, 502285 India; 2 Department of Computer Science and Engineering, JAIN (Deemed-to-Be University), Bengaluru, 562112 India; 3 Department of Computer Science and Engineering, Sathyabama Institute of Science and Technology, Jeppiar Nagar, Chennai, 600119 India; 4 School of Computer Science Engineering & Information Systems(SCORE), Vellore Institute of Technology (VIT), Vellore, 632014 Tamil Nadu India; 5 School of Science Engineering and Environment, University of Salford, Manchester, UK; 6 Department of Electrical and Computer Engineering, Lebanese American University, Byblos, Lebanon; 7 College of Computer Sciences and Information Technology, King Faisal University, 31982 Hofuf, Saudi Arabia; 8 Department of management, College of Business Administration, Princess Nourah Bint Abdulrahman University, P.O. Box 84428, 11671 Riyadh, Saudi Arabia

**Keywords:** Federated learning, Artificial intelligence of medical things, Healthcare systems, Device categorization, Device labeling

## Abstract

Categorizing Artificial Intelligence of Medical Things (AIoMT) devices within the realm of standard Internet of Things (IoT) and Internet of Medical Things (IoMT) devices, particularly at the server and computational layers, poses a formidable challenge. In this paper, we present a novel methodology for categorizing AIoMT devices through the application of decentralized processing, referred to as "Federated Learning" (FL). Our approach involves deploying a system on standard IoT devices and labeled IoMT devices for training purposes and attribute extraction. Through this process, we extract and map the interconnected attributes from a global federated cum aggression server. The aim of this terminology is to extract interdependent devices via federated learning, ensuring data privacy and adherence to operational policies. Consequently, a global training dataset repository is coordinated to establish a centralized indexing and synchronization knowledge repository. The categorization process employs generic labels for devices transmitting medical data through regular communication channels. We evaluate our proposed methodology across a variety of IoT, IoMT, and AIoMT devices, demonstrating effective classification and labeling. Our technique yields a reliable categorization index for facilitating efficient access and optimization of medical devices within global servers.

## Introduction

Internet of things (IoT) have turn into the most important part of our day to day life due to the rise in the technology and it is in continues process of advancement. Humans are dependent on internet which leads to the rapid growth in the research area of IoT, Artificial Intelligence (AI) where the human expectations are understood by the machines automatically, machines are developed in such a way that it predicts, classifies and provides a reliable decision making using the in-built algorithms and advanced training datasets. Artificial Intelligence (AI) is considered to be one of the traditional method to generate the data throughout global IoT devices. AI advancement is started in the Deep learning (DL) and Machine Learning (ML) for the purpose of designing models to train data on growing demand of the applications on intelligent IoT in various fields like smart healthcare, smart vehicles and smart cities [1].

The convergence of IoT and AI in the medical field has ushered in a new era of innovation and transformation. Through the seamless integration of connected devices and advanced analytics, healthcare providers can now harness the power of real-time data insights to proactively address health concerns and prevent adverse outcomes. With the rise of IoT-enabled medical devices, the concept of IoMT has emerged as a specialized domain dedicated to leveraging interconnected technologies for healthcare applications. This paradigm shift has paved the way for AIoMT, where Artificial Intelligence augments the capabilities of IoMT devices, enabling more sophisticated analysis and prediction of health-related issues. However, traditional approaches to AI data processing face challenges in the medical context, where privacy and security concerns are paramount. Recognizing this, Federated Learning has emerged as a promising solution. By decentralizing the training process and allowing devices to collaboratively learn from local data without sharing sensitive information, FL ensures confidentiality while still enabling robust AI models to be developed [2].

In the rapidly evolving landscape of smart healthcare, Federated Learning has garnered significant attention for its ability to reconcile the need for data-driven insights with the imperative of safeguarding patient privacy. As healthcare data continues to proliferate at an unprecedented rate, innovative solutions like FL are essential for unlocking its full potential while ensuring compliance with regulatory standards and ethical principles [3]. As we look ahead, the synergy between IoT, AI, and Federated Learning holds immense promise for revolutionizing healthcare delivery and improving patient outcomes. By harnessing the collective intelligence of interconnected devices in a secure and privacy-preserving manner, we can unlock new frontiers in personalized medicine, disease prevention, and population health management.

In the realm of medical healthcare, where the sensitivity of data is paramount for accurate prediction and detection of diseases, the use of Federated Learning (FL) emerges as a crucial safeguard. FL ensures that raw data remains shielded from third-party access or leakage, thereby enhancing data privacy—a critical consideration in healthcare contexts [4]. FL also contributes to the optimization of network communication by reducing latency, particularly in scenarios where transmitting IoT data to centralized servers may encounter delays. By distributing the training process across localized nodes, FL conserves network resources, facilitating more efficient data training operations. The FL enhances the quality of learning by accelerating the training process and achieving higher accuracy rates.

This improvement is particularly notable when compared to traditional AI approaches, which may struggle to attain optimal performance due to limitations in available data and computational resources. Given these unique characteristics, Federated Learning has emerged as a cornerstone of innovation in medical applications. Its ability to address the challenges of data sensitivity, network efficiency, and learning quality positions FL as a preferred solution for realizing the full potential of IoT and AI in advancing healthcare outcomes. As the adoption of FL continues to expand, we can anticipate transformative advancements in medical diagnosis, treatment optimization, and patient care delivery.

## Literature reviews

Development in the research area of federated learning is due to its high performance in the communication and efficiency in a star network for distributing efficient training data it has created an open challenge and opportunity for the trending research on the medical health with AI field. Data privacy is the biggest challenge in the field of medial health care, it is important to secure the data and avoid the miss use of the data at any instant of time to update this challenge federated learning is introduced merging with AI [5]. A model based on federal learning was designed to simplify and maintain the original perverse of the trained data between the circulated users in order for the advancement of applications in the healthcare systems [6]. The model was designed with two period contracts which give the performance of self-revealing with high profit when compared to other uniform schemes. Further the model was purposed to work in future with the reinforcement learning. The overall model was based on edge computing using FL system.

In this study [7], the researchers have made a detailed study on federal learning for three cloud-edge-devices using the edge devices like smart-phones. The model restricts the usage of undesirable data and saves the storage and transmission of the data in the cloud platform. For the purpose of reducing the timing of interaction and usage of heavy data edge computing is come into existence it performs well when compared to standard cloud based approach. It is predicted by this study that in industry using Internet there will be soon 4.0 contexts based on edge based approach along with the FL. In this study [8], the researcher proposed a model which is suited best for the human centric application in the healthcare industry, the main benefit of this software reduced the data processing and transmitting time for various real time implementation on hardware. The study defines Body-Edge as a valid and reasonable cost dependent application used in healthcare. Due to the rapid increase in the use of internet in technology, maintains of accuracy with the data privacy has become one of the open challenge for the researches. Data sustainability is also considered to be the most important parameter. To overcome this health care monitoring system has adopted machine learning along with FL to except the challenge [9–14]. A model is designed with a deep federal learning for the health care system for the purpose of data monitoring using IoT devises. It also aimed to predict the skin disease in human. The results were obtained in the form of 97% improvisation of area under curve (AUC) [15].

The recommendation models on resources and services are based on the process and techniques as discussed in [16] is intended to provide a customizable lifestyle of the users Whereas the services provided via 5G and 6G is thus dependent on multiple features and internally improve the quality of services as discussed in [17, 18]. The 5G cloud networking model recommends the data services as per the mode of application demand. Typically, the recent advancements from the IoMT and IoT is recorded in [19–22] further discussing the influence and challenges in implementing the recommendation models. The extended technological development from augmented AI is discussed in [23] to support the recommendation framework of IoMT devices in smart cities infrastructure. The articles [24–26] discusses the applications and possible recommendations on IoMT devices.

## Methodology

**Fig. 1:**
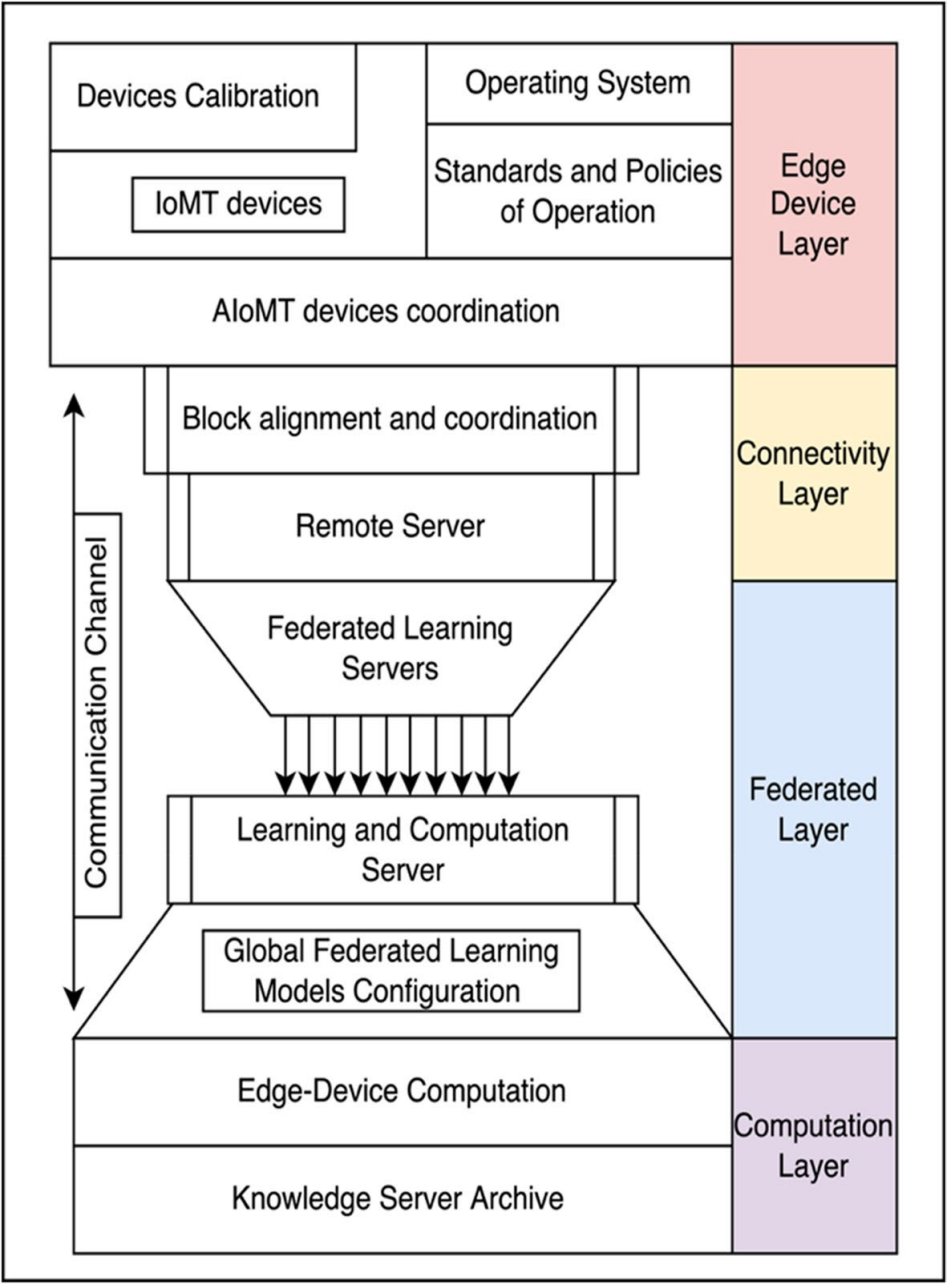
Proposed system architecture

The proposed methodology is based on the inclusion of smarter and efficient classification on AIoMT based medical devices via federated learning models. The proposed model is developed and designed on the primary principles of IoT and IoMT devices with an improved version of stabilization under categorization. The proposed system is represented in Fig. 1 under four major layer distribution function. The primary layer is the Edge device layer, the second is connectivity layer, third is federated layer and fourth is computation layer. The orientation of these layers is to build a sustainable product for categorizing the devices based on the user demand and request [13]. The streamlining of data is developed using AIoMT device coordination to assure the devices are authentic and have privilege to participate in federated based distributive learning environment. The proposed system is aligned on a connectivity layer under a third party communication channel such as 4G, LTE, 5G and 6G systems. The remote server acts as an incubator to resolve the occurrences of multiple device registrations and instances. The process of federated learning is developed and streamlined with a hand-shake property to exchange data and information via a federated learning agreement to train and validate the model datasets [14]. The federated servers are connected and assures the data integrity is maintained via multiple scenarios of training and computation process.

**Fig. 2:**
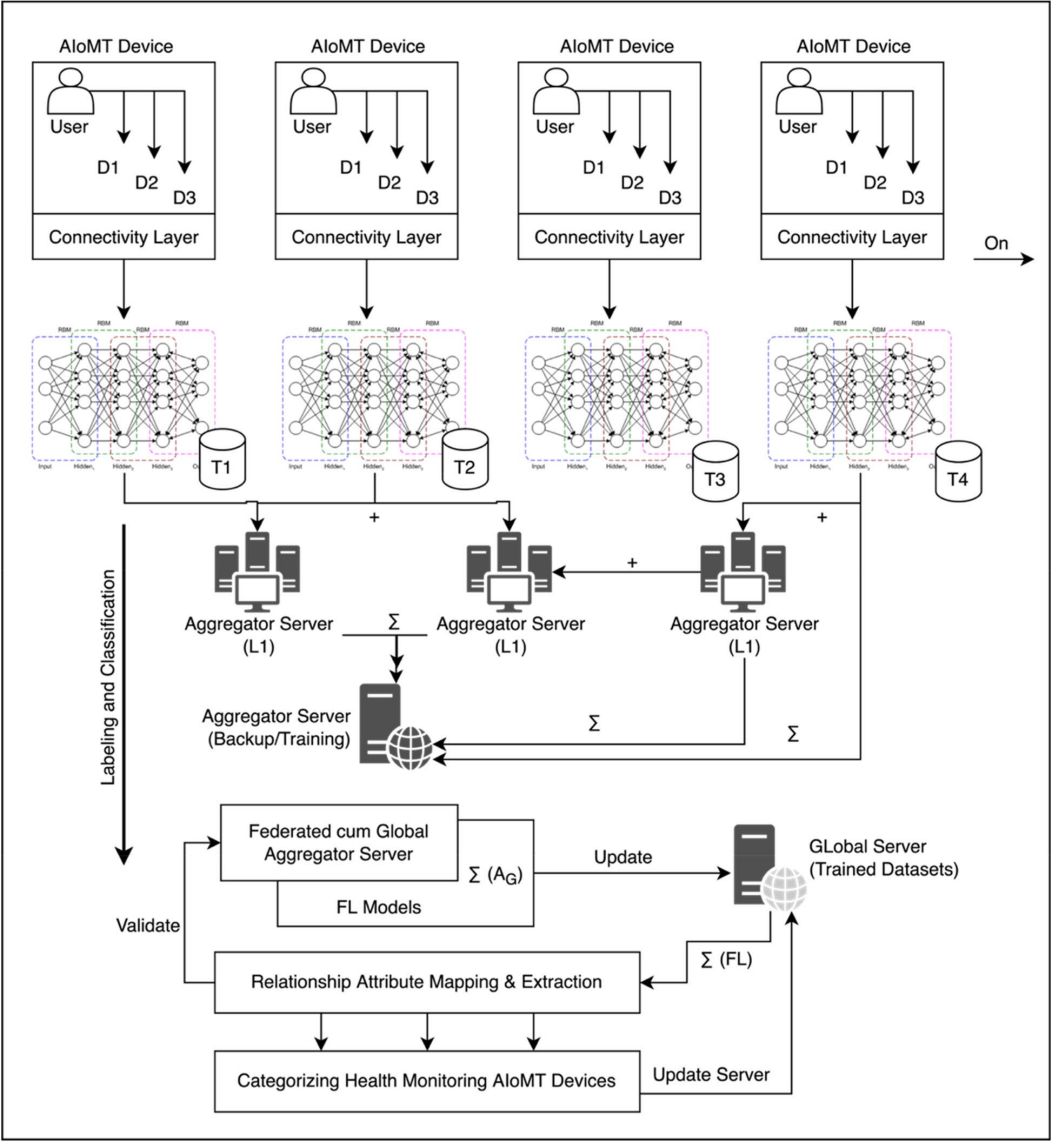
AIoMT based categorization using federated learning models

The process of federated learning is summarized in a detailed manner in Fig. 2. The representation is demonstrated from an initial user to device relationship mapping. The users and its dedicated devices are processed via a trained neural networking algorithms to fetch responsive information without downloading the data in a centralized or global server. The process of learning from individual devices is automatically updated to the reporting aggregator server [15]. The global server is based on the synchronization and processing a relationship mapping for IoMT and AIoMT devices. The FL models are sophisticated in extracting information based on recommended device patterns. These patterns provide a decision making in categorizing the health monitoring devices via a labeling operation under dynamic structure.

## Edge devices layer

The computational requirements of Artificial Intelligence based IoMT devices is a decentralized server processing and analytics in gathering the needful information. The health monitoring devices are customized with respect to device initial configuration and parameters. Consider the local devices as $\left( D \right)$ with initial configuration towards IoT operating standards and $\left( {D_{m} } \right)$ are devices with IoMT standards of operation. The consideration $\left( {D_{m} \subseteq D} \right)$ is aligned within a series of operating framework standards. Consider the configuration as $\left( {C_{f} } \right)$ aligned with $\left( {D_{m} } \right)$ devices as $\left( {C_{f} = C_{f1} ,C_{f2} ,C_{f3} ....} \right)$ with $\forall C_{fn} \Rightarrow \sum D_{m}$. The n^th^ devices added into this framework is correlated and expanded in a series of coordinated as shown in Eq. 1

1 $$C_{f\left( G \right)} = \mathop {\min }\limits_{n} \left( {\sum\limits_{i = 1}^{n} {\left( {D_{m} } \right)_{i} \frac{{\delta \left( {C_{f} } \right)_{i} }}{\delta t}} } \right)$$

2 $$C_{f\left( G \right)} = \mathop {\min }\limits_{n \to \infty } \left( {\sum\limits_{i = 1}^{\infty } {\sum\limits_{j = j + 1}^{n} {\frac{{\delta \left( {D_{m} } \right)_{i} \to \delta \left( {C_{f} } \right)_{i} }}{\delta t}} } } \right)$$

According to Eq. 2, the principle compound of configuration server is aligned and coordinated with devices $\left( {D_{m} } \right)$ in services of medical applications. Thus the primary evaluation of configuration management with reference to $\left( {D_{m} } \right)$ is extracted in Eq. 2.

### AIoMT Device Coordination

The functional devices in a given $\left( {D_{m} } \right)$ set are extracted as $\left( {D_{k} } \right)$ as $\left( {D_{k} \Rightarrow \left[ {\left( {D_{m} - D} \right) \cup D_{A} } \right]} \right)$ where $\left( {D_{A} } \right)$ is the active set of IoMT devices. The functional/active devices are programmed and continued with inter-attribute co-relationship mapping. Consider the attribute $\left( A \right)$ in $\left( {D_{A} } \right)$ devices as $\left( {D_{A} \to A_{1} ,A_{2} ,A_{3} ....A_{n} } \right) \Rightarrow f\left( {A_{{D_{A} }} } \right)$. The matrix of evaluation and attribute mapping $\left( {A_{m} } \right)$ is attained and mapped as shown in Eq. 3

3 $$f\left( {A_{m} } \right) = \mathop {\lim }\limits_{n \to \infty } \left( {\sum\limits_{i = 0}^{n} {\left( {f\left( {A_{n} } \right)_{{D_{A} }} } \right) \cdot dt} } \right)$$

4 $$\therefore f\left( {A_{m} } \right) = \mathop {\lim }\limits_{n \to \infty } \left( {\sum\limits_{i = 0}^{\infty } {\prod\limits_{j = i + 1}^{n} {\left[ {\frac{{\partial \left( {D_{A} } \right)_{i} }}{\partial t} \to \frac{{\partial \left( {A_{n} } \right)_{j} }}{\partial t}} \right]} } } \right)$$

5 $$\therefore f\left( {A_{m} } \right) = \mathop {\lim }\limits_{n \to \infty } \left( {\sum\limits_{i = 0}^{\infty } {\prod\limits_{j = i + 1}^{n} {\left[ {\frac{{\partial \left( {D_{A} } \right)_{i} }}{\partial t} \to \left\{ {\frac{{\partial \left( {A_{n} } \right)_{j} \cup \partial \left( {C_{f} } \right)_{j} }}{\partial t}} \right\}} \right]} } } \right)$$

The evaluation with reference to Eqs. 4 and Eq. 5 demonstrate the relevance of multidimensional attribute mapping with $\forall \left( {A_{n} } \right)_{j} \Rightarrow \forall \left( {C_{f} } \right)_{j}$ a generalized mapping order. Typically the configuration vector $\left( {C_{f} } \right)_{\left[ G \right]}$ is resultant product for accessing values based attributes as $\left( {A_{j} } \right) \Rightarrow \sum \left( {A_{j} \cup \left( {D_{A} } \right)_{j} } \right)$ shown in Eq. 6

6 $$\sum \left( {f\left( {A_{m} } \right)} \right) = \frac{{\log \left( {D_{A} } \right)}}{{\left( {C_{f} } \right)_{\left[ G \right]} }}\left( {\sum\limits_{i = 1}^{\infty } {\sum\limits_{j = i + 1}^{n} {\frac{{\partial \left( {D_{A} } \right)_{i} \to \partial \left( {f\left( {A_{m} } \right)_{j} } \right)}}{\partial t}} } } \right)$$

The progression function is aligned and mapped with a self-archive based iteration as shown in Eq. 6, where $\forall \left( {\sum \left( {f\left[ {A_{m} } \right]} \right)} \right)$ is a dependent variable on $\left( {D_{A} } \right)$ based AIoMT devices. The backdrop assumption extracts the parameters such as trained datasets of individual devices. These hidden logs of trained dataset are processed and aligned using federated learning terminologies for effective processing.

### IoMT device standards and policies alignment

The considered devices for processing and computation is based on the policies and operating principles of IoT and IoMT. The set of medical devices are categorized as a resultant matrix with sensitive data processing and calibration. With the inclusion of artificial intelligence, the IoMT based devices are further re-configured to map the service standards and termed as AIoMT devices. The basic principles are further considered for representation purpose. The operating principles are limited and progress under sensitive data transfer and exchange principles. The proposed framework is extended with federated learning framework. The federated learning (FL) is defined on decentralized data alignment and coordination principles of operations. The requirement of federated learning system is fetched from remote data point for AIoMT device connectivity and communication.

## Connectivity layer

The coordination principle of AIoMT devices with reference to federated learning is retrieved for the process of communication. The connectivity layer is an inclusion of block alignment coordination and remote server connectivity as shown in Fig. 1. The main contribution of connectivity layer is to fetch the interconnected AIoMT devices trained dataset with respect to the operating locations. Consider the process of extracting into a decentralized manner as projected below.

## Block alignment and federated learning model

Consider the incoming block of data chain from the shortlisted and approved AIoMT devices as $\left[ {\left( B \right)_{{D_{A} }} } \right]$ with each block is aligned to the matrix index (h) as $\left[ {h_{i} \Rightarrow \left( B \right)_{{D_{A} }} } \right]$. $\left[ {\forall f\left( {A_{m} } \right) \Rightarrow \sum \left( B \right)_{{D_{A} }} } \right]$ is aligned with reference to individual block size and occurrence ratio $\left( {O_{c} } \right)$ as $\left[ {\left( B \right)_{{D_{A} }} \Rightarrow \partial \left( t \right).O_{c} } \right]$ with instance of incoming data-blocks AIoMT is fragmented and realigned as shown in Eq. 7

7 $$\left[ {\left( B \right)_{{D_{A} }} } \right] = f\left( {A_{m} } \right).\partial \left( {O_{c} } \right)_{k} .dt$$

8 $$\therefore \left[ {\sum \left( B \right)_{{D_{A} }} } \right] = f\left( {A_{m} } \right).\sum\limits_{i = 0}^{n} {\frac{{\partial \left( {O_{c} } \right)_{k} }}{\partial t}} \int\limits_{0}^{{D_{A} }} {\left( h \right)_{{O_{c} }} }$$

With reference to Eq. 8, the relevance of each incoming block is aligned and categorized with respect to $f\left( {A_{m} } \right)$ and the occurrence ratio $\left( {O_{c} } \right)$ with respect to matrix index (h). The relevance of each block of data is further evaluated under a schematic representation of secondary blocks as shown in Eq. 9

9 $$\left[ {\sum \left( B \right)_{{D_{A} }} } \right] = \sum\limits_{i = 0}^{\infty } {\prod\limits_{j = i + 1}^{n} {\left( {\frac{{\delta \left( {f\left( {A_{m} } \right)_{i} } \right)}}{\delta t} \cup \frac{{\delta \left( {\sum \left( B \right)_{{D_{A} }} } \right)_{j} }}{\delta t}} \right)} }$$

10 $$\therefore \left[ {\sum \left( B \right)_{{D_{A} }} } \right] = \mathop {\lim }\limits_{n \to \infty } \left[ {\sum\limits_{i = 0}^{\infty } {\prod\limits_{j = i + 1}^{n} {\left( {\frac{{\delta \left( {f\left( {A_{m} } \right)_{i} } \right) \cup \delta \left( {\sum \left( B \right)_{{D_{A} }} } \right)_{j} }}{\delta t}} \right)} } } \right]$$

The functional parameters of Eq. 10 are cumulative approach of connectivity layer in building up the relevance of each inter-related blocks from AIoMT devices. The further step is to include the remote server communication and path alignment. The relevance of server (S) under the connection (C_Conn_) is described as shown in Eq. 11 with weight (W) matrix.

11 $$S = W_{t} + D_{A} .\eta .h_{i}$$

12 $$\sum S = W_{t} + \sum D_{A} .\eta .h_{i} + W_{t + i}$$

where, the relevance of server (S) is built and navigated within the matrix index $\left( {h_{e} } \right)$ for constant and represented values in weight $\left( {W_{t} } \right)$ and $\left( {W_{t + i} } \right)$ as relevance. $\left( \eta \right)$ is used to represent and process the binding probabilities of devices in active state to avoid unambiguous representations. These active state devices $\left( {D_{A} } \right)$ are primarily filtered as non-operational active devices as shown in Eq. 13

13 $$f\left( \eta \right) = \mathop {\lim }\limits_{n \to \infty } \left( {h_{i} .\sum \left( {D_{k} \cap D_{A} } \right) - \sum \left( {D_{k} } \right)} \right)$$

The binding probability $\left( \eta \right)$ is used to re-align and categorize the devices and its interconnected relevance with weights $\left( W \right)_{t}^{\infty } \to \partial \left( {W_{t} } \right) \Rightarrow \left. {D_{A} } \right)_{0}^{\infty }$. The ratio assures the process is streamlined and customized for higher order relevance.

The prospective of this research article is to categorize health monitoring devices under AIoMT operating standards. The federated learning based AIoMT ecosystem is defined as Fig. 2. The model assures the operation of an individual device connected and operated by a single user and group under the mode of usage as user $\left( {U \to U_{1} ,U_{2} ,U_{3} ....U_{n} ....\infty } \right)$ and devices $\left( {U_{i} \to D_{1} ,D_{2} ,D_{3} .....D_{k} ......D_{A} .....D} \right)$ where each operating device is consolidated with an independent user $U$. The process assures a reliable mode of dependencies with each independent attribute to build a local computational unit as shown in Eq. 14.

14 $$\left( {F_{L} } \right)_{L1} = \sum\limits_{i = 0}^{\infty } {\sum\limits_{j = 1}^{(n,i)} {\left[ {f\left( U \right)_{i} \to \sum\limits_{k = 0}^{n} {\left( {\frac{{\partial \left( {D_{j} } \right)}}{\partial t} \cup \left( {L_{1} } \right)_{k} } \right)} } \right]} }$$

Where each passing elements $\left( {L_{1} } \right)_{k}$ layers in federated learning ecosystem is relatively captured and processed to function as primary or orbiter layer in customizing federated learning models as shown in Eq. 15

15 $$\sum \left( {F_{L} } \right)_{L2} = \sum\limits_{i = 0}^{\infty } {\prod\limits_{j = i + 1}^{n} {\left( {\mathop {\lim }\limits_{n \to \infty } \left[ {f\left( U \right)_{i} \to f\left( \eta \right)_{j} } \right] \cup \left( {F_{L} } \right)_{L1} } \right)} }$$

16 $$\therefore \sum \left( {F_{L} } \right)_{L2} = \sum\limits_{i = 0}^{\infty } {\prod\limits_{j = i + 1}^{n} {\left( {\left( {\left( {F_{L} } \right)_{L1} } \right) \Leftrightarrow \mathop {\lim }\limits_{n \to \infty } \left[ {f\left( U \right)_{i} \to f\left( \eta \right)_{j} } \right]} \right)} }$$

According to Eq. 16, the relevance of multiple occurrences in the existing AIoMT system is programmed and customized towards rational building blocks of second layer federated layer $\left( {L2} \right)$ with $\left[ {\left( {F_{L} } \right)_{L1} \subseteq \left( {F_{L} } \right)_{L2} } \right]$ in accordingly to the primary data managed and processed for customizing privileges in IoMT to AIoMT enabling devices. The relevance continues in according to n^th^ layer of federated learning models as shown in Eq. 17 and 18 respectively.

17 $$\therefore \sum \left( {F_{L} } \right)_{Ln} = \int\limits_{0}^{\infty } {\int\limits_{n} {\left. {\left( {\left( {F_{L} } \right)_{Li} } \right)} \right]_{0}^{\infty } } } \cup \left\{ {\sum\limits_{i = 0}^{\infty } {\prod\limits_{j = i + 1}^{n} {\left( {\mathop {\lim }\limits_{n \to \infty } \left[ {\partial \left( {\frac{{f\left( U \right)_{i} \to f\left( \eta \right)_{j} }}{t}} \right)} \right]} \right)} } } \right\}$$

18 $$\therefore \sum \left( {F_{L} } \right) = \left. {\left( {F_{L} } \right)} \right]_{0}^{\infty } \cup \left\{ {\sum\limits_{i = 0}^{\infty } {\prod\limits_{j = i + 1}^{n} {\left( {\mathop {\lim }\limits_{n \to \infty } \left[ {\partial \left( {\frac{{f\left( U \right)_{i} \to f\left( \eta \right)_{j} }}{t}} \right)} \right]} \right)} } } \right\}$$

Where $\left( {F_{L} } \right)$ is the cumulative representation of federated layer and user data corelationship, the functional parameter of developing federated layer is to develop a reliable data understandability network. The aggregation server plays a major role for user data synchronization as shown in Eq. 19

19 $$S_{A} = W_{t} + \sum \left( {\Delta F_{L} } \right)_{0}^{\infty } .f\left( \eta \right).h_{i} + W_{t + 1}$$

Equation 19 is reframed form the primary server alignment principle as shown in Eq. 12 with relevance to the replacement of federated layer $\left( {\Delta F_{L} } \right)$ to device $\left( {D_{A} } \right)$ under the $\left( \eta \right)$ probabilities matrix as shown in Eq. 20.

20 $$\sum S_{A} = \sum W_{t} + \mathop \sum \limits^{\infty } \left( {\Delta F_{L} } \right).f\left( {\eta_{i} } \right).h_{i} + \sum W_{t + 1}$$

Where Eq. 20 assures the information is synchronized and customized with relevance to the federated learning model and a trained data $\left( {\Delta Tr} \right)$ is generated with relevance to $\sum S_{A}$ server’s synchronization. The federated cum global aggregator server $\left( {A_{G} } \right)$ is used to consolidate and restructure the paradigms of $\sum S_{A}$ as shown in Eq. 21

21 $$\sum \left( {A_{G} } \right) = \sum\limits_{i = 0}^{\infty } {\prod\limits_{j = i + 1}^{n} {f\left( \eta \right)_{i} \left[ {\frac{{\partial \left( {S_{A} } \right)_{i,j} }}{\partial t}} \right]} }$$

Where the synchronized data stream of $\left( {A_{G} } \right)$ is represented and customized with reference to globally connected servers $\sum S_{A}$ with each probability $\left( \eta \right)_{i}$ is restructured to frame a relevant bounding in data acquired. The global aggregation server $\sum \left( {A_{G} } \right)$ is further evaluated with relevance to mapping of attributes for effective customization and categorization of devices using AIoMT. Typically the federation learning models are synchronized and communicated via third party communication channel and bandwidth spectrum of 3G/LTE/4G and proposed 5G. The inter-relevance can be shown as Eq. 22

22 $$C_{Conn} = \mathop {\lim }\limits_{n \to \infty } \left( {\sum\limits_{i = 1}^{\infty } {f\left( {A_{G} } \right)_{i} \times \left( {\Delta Tr} \right)_{{i \to \sum S_{A} }} } } \right)$$

23 $$\therefore C_{Conn} = \frac{{\Delta Ther\left( {\arg \min \left( {D_{A} } \right)} \right)}}{D}\left( {\sum\limits_{i = 1}^{\infty } {f\left( {A_{G} } \right)_{i} \times \left( {\Delta Tr} \right)_{{i \to \sum S_{A} }} } } \right)$$

According to Eq. 23 the connection and relevance of each incoming connection is coordinated at $\sum \left( {A_{G} } \right)$ under aligned $\left( {\Delta Tr} \right)$ training set $\left( {\forall Tr \to S_{A} } \right)$ at an interdependent matrix exchange ratio. The process of AIoMT devices are cumulatively coordinated and updated.

## Computational layer

The computational layer is the inbuilt and self-archiving support for the computation of training datasets $\left( {\Delta Tr} \right)$ in the overall synchronized state of operation. The layer extracts information from a layer $\sum \left( {A_{G} } \right)$ server as $\left[ {\sum \left( {A_{G} } \right) \Rightarrow \sum \left( {S_{A} } \right)_{0}^{n} } \right]$ and the training dataset $\left( {\Delta Tr} \right)$ needs a regular updating. The extracted federated later study and training in further synchronization with global servers of training datasets.

The process of categorization is supported and streamlined as $\left( {\Delta {\mathbb{R}}} \right)$ for an initial categorization with $\left( \xi \right)$ as each set of customized devices as $\left( {\xi = D_{1} ,D_{2} ,D_{3} .....D_{n} } \right)$ such that $\left( {\xi \subseteq D \subseteq D_{k} \subseteq D_{A} } \right)$. The independent variables in $\left( {D_{A} \Rightarrow \xi_{A} } \right)$ with all coordination $\left( \xi \right)_{{\left[ {A,k} \right]}}$ in generalized categorization as $\left( {\forall {\mathbb{R}}_{\xi } \Rightarrow \xi_{{\left[ {A,k} \right]}} } \right)$ such that $\left( {\forall {\mathbb{R}}_{\xi } \Rightarrow \Delta Tr_{\xi } } \right)$, such that $\left( {\Delta Tr_{\xi } } \right)$ is the categorized training set in decentralized servers as shown in Eq. 24.

24 $$\Delta {\mathbb{R}} = \left[ {f\left( {S_{A} } \right) \cup f\left( {A_{G} } \right)} \right]_{0}^{\infty } - \sum\limits_{i = 0}^{\infty } {\left( {D_{i} } \right)}$$

Where the AIoMT devices $\left( {D_{i} } \right)$ are labeled based on the performance vector and integrity factor for relevance mapping in categorization. The customized devices $\left( \xi \right)$ are further categorized as shown in Eq. 25.

25 $$f\left( {\Delta {\mathbb{R}}} \right) = \sum\limits_{i = 0}^{\infty } {\sum\limits_{j = i + 1}^{n} {\left( {\xi_{{\left[ {Ai,kj} \right]}} \Rightarrow \frac{{\partial \left( {D_{A} } \right)_{i} \cup \partial \left( {D_{k} } \right)_{j} }}{\partial t}} \right)} }$$

26 $$\therefore \sum \left( {\Delta {\mathbb{R}}} \right) = \sum\limits_{i = 0}^{\infty } {\sum\limits_{j = i + 1}^{n} {\left( {\xi_{{\left[ {Ai,kj} \right]}} \cup \left( {\frac{{\partial \left( {D_{A} } \right)_{i} \cup \partial \left( {D_{k} } \right)_{j} }}{\partial t}} \right)} \right)} }$$

According to Eq. 26, the customization of devices (AIoMT) is functionally extracted. The categorization assures that the devices are streamed into a valid buffer zone for labeling the categories. This improves the ratio of understanding and validation with respect to device validation and its labeling order.

## Results and Discussions

**Fig. 3:**
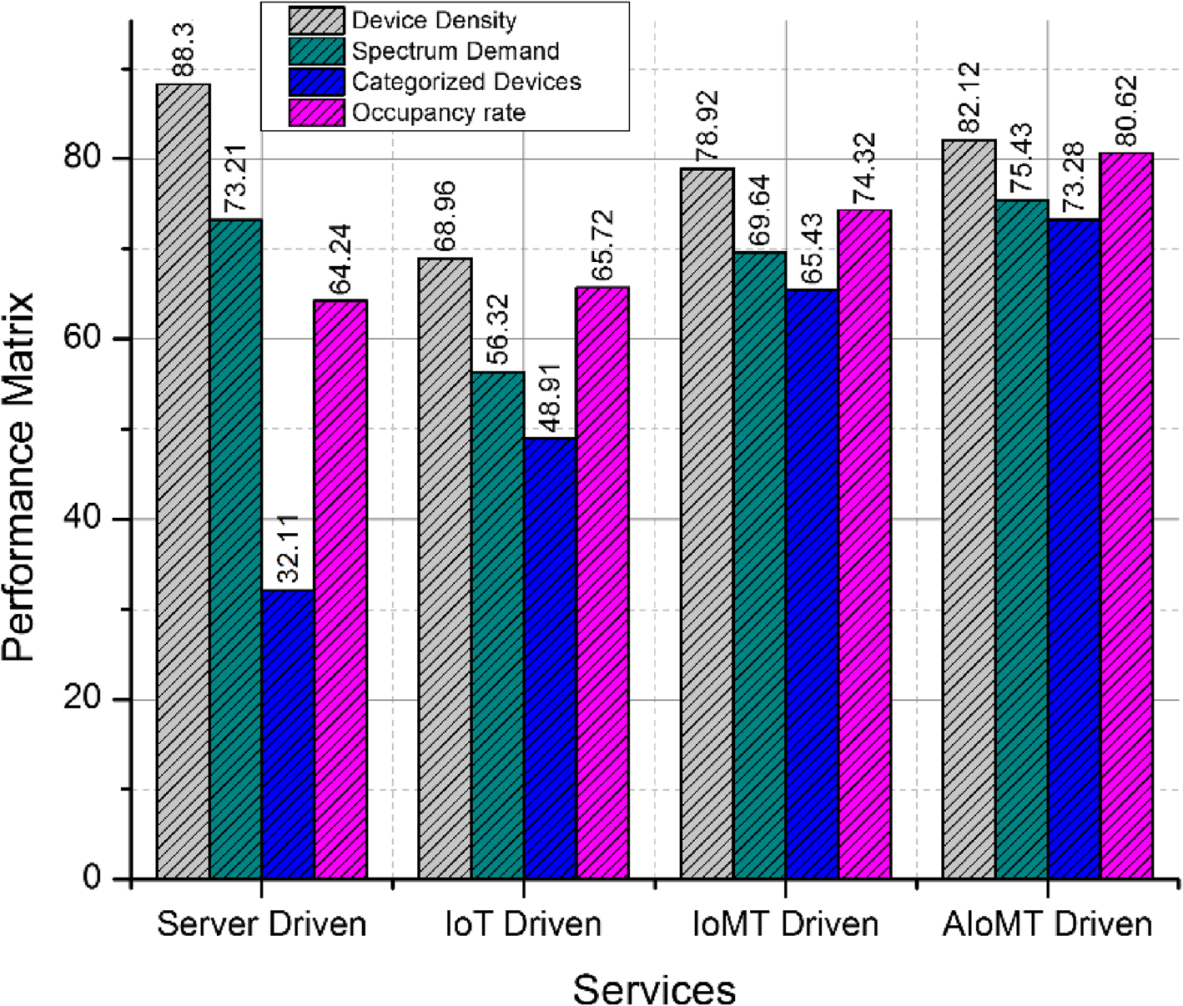
Devices behaviors analysis and performance matrix

The proposed technique has categorized the IoMT based devices under the influential representation of AIoMT. The process is lined in accordance to the IoT operating standards and principles. The proposed technique has retrieved an attribute matrix as shown in Table 1. The attribute matrix projects has a detailed overview with respect to the relevance in various services provided by medical applications via server driven typical systems, IoT, IoMT and proposed AIoMT framework. The categories such as general purpose, medical purpose and emergencies purpose are streamlined to understand the behavior pattern of various devices in these multiple frameworks. The representation of device behavior and performance paradigms is represented in Fig. 3. The process validates the demand density and spectrum representation via various services such as server driven applications, IoT, IoMT and proposed AIoMT framework. The resultant matrix has shown a higher performance ratio compared to the existing process as the AI driven categorization is customized under occupancy rate. The overall recommendations on $\left( {D_{i} } \right)$ devices have resulted in streaming $\left( \xi \right)$ customized devices in AIoMT environment.

**Table 1:** Attribute matrix and labeling paradigms comparative representation

Device Type	Device Category	Labeling Status	Attributes Mapped	status mapping	retrieval strength
**Server driven**	General	Nill	No	NA	low
	Medical	Nill	No	NA	low
	Emergencies	Yes	Yes	NA	medium
**IoT Driven**	General	Nil	Yes	Yes	medium
	Medical	Yes	No	No	low
	Emergencies	Yes	No	No	low
**IoMT driven**	General	Yes	No	No	low
	Medical	Yes	Yes	Yes	medium
	Emergencies	Nill	No	No	low
**AIoMT driven**	General	Yes	Yes	Yes	medium
	Medical	Yes	Yes	Yes	high
	Emergencies	Yes	No	No	low

**Fig. 4:**
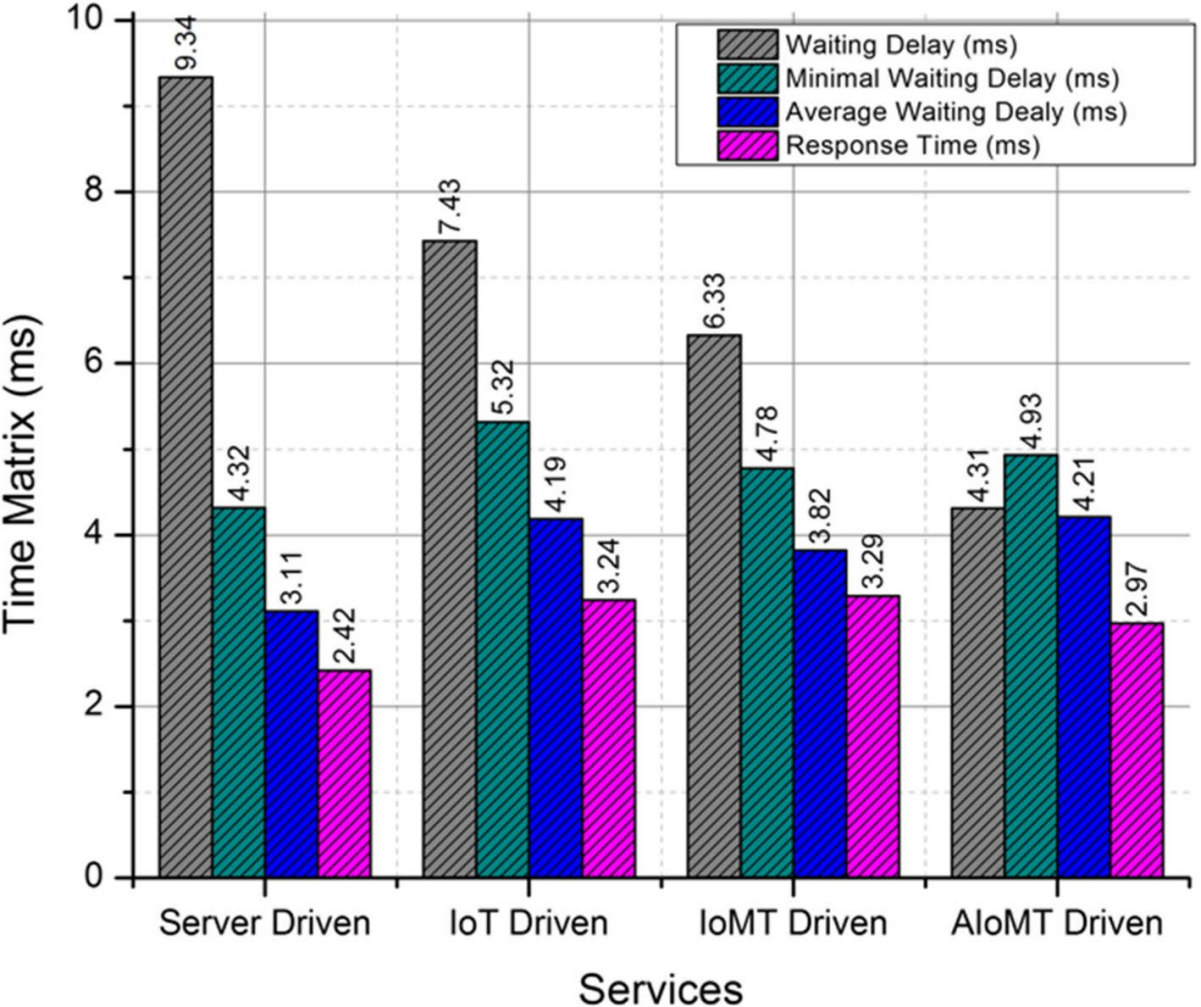
Time matrix evaluation and performance enhancement

The evaluation of delay and time management is studied and observed in the experimental setup provided by the proposed AIoMT technique. The time frame parameters such as waiting delay, minimal waiting time, response time and average waiting time is computed. The process assures a reliable time management series compared to the existing services and platforms for labeling the devices. The orientation has minimized the waiting delay with 38.34% compared to trivial IoMT connected servers. The labeling in these servers is pre-trained and hence the dynamic allocation and fragmentation of information via user request is not monitored. Overall the proposed AIoMT based approach is out-performing the existing systems with a saturated time delay management under a consistent evaluation process. The Fig. 4 depicts time matrix evaluation and performance enhancement.

**Fig. 5:**
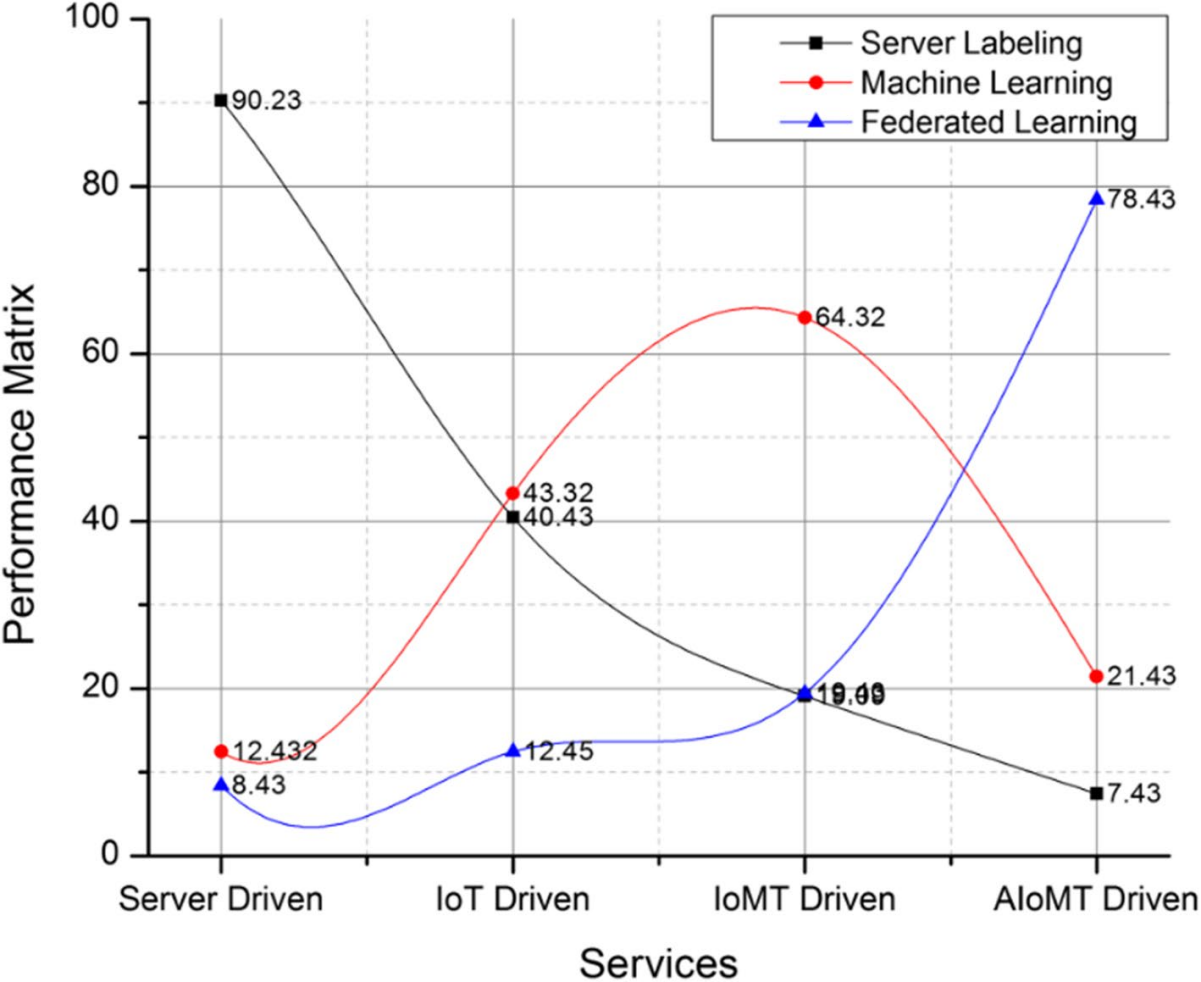
Comparative analysis of performance matric v/s technological platforms

**Fig. 6:**
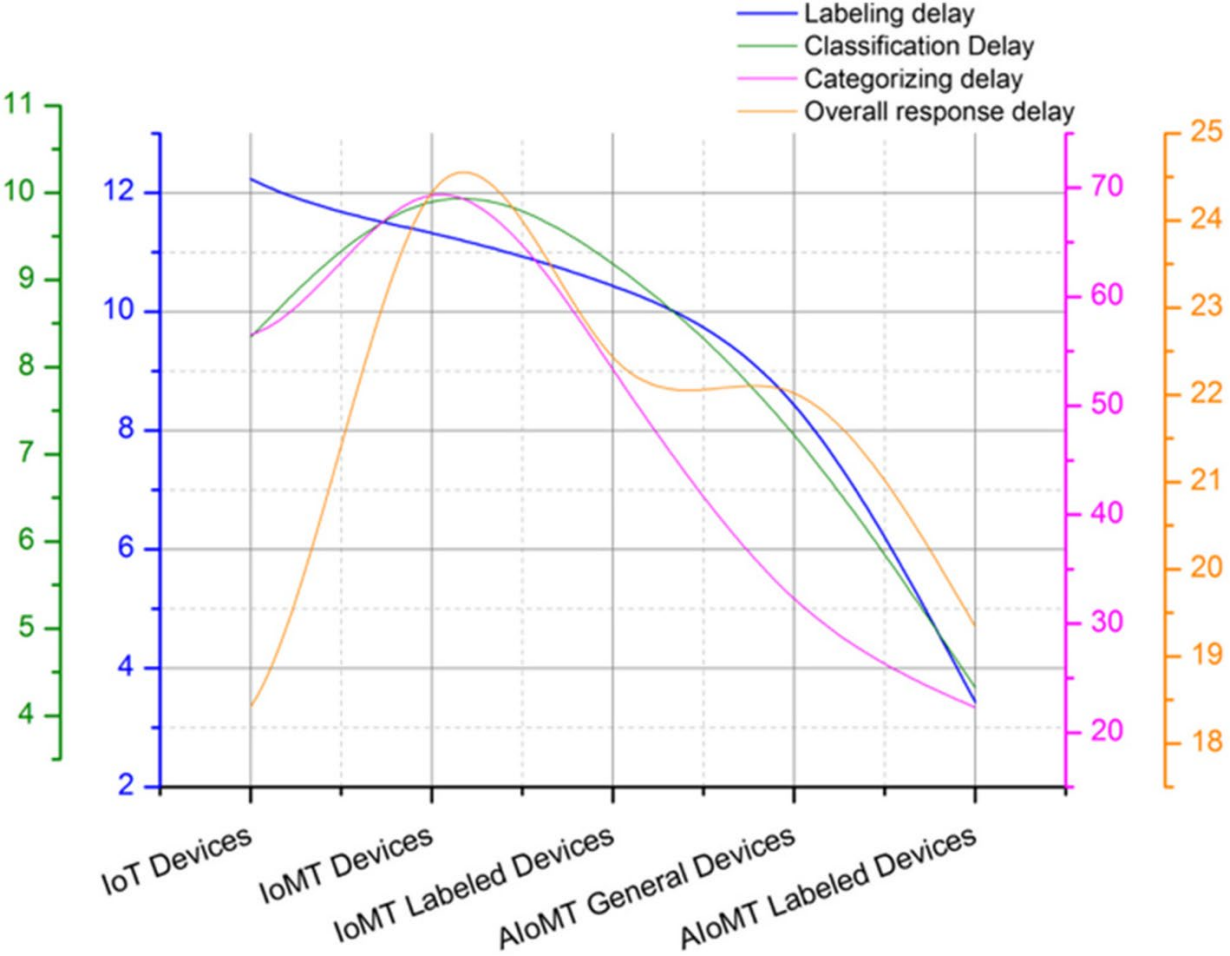
Categorization performance matrix representation

The performance matrix of various comparative algorithms is computed in Fig. 5 with reference to server labeling, machine learning based labeling in IoT and AIoMT devices and proposed federated learning based labeling. The process of federated learning is implemented in a minimal responsive manner compared to the other trivial approaches with a gradual incline on appending federated learning based labeling feature in the IoMT and AIoMT devices. Overall the proposed system has restricted the labeling based on machine learning and server based to minimize the computation delay as demonstrated in Table 2 and Table 3 respectively. The process of delay computation is monitored on representation of various categorization parameters such as labeling delay, classification delay, categorization delay and overall response delay. A detailed representation of categorization and labeling on AIoMT devices is demonstrated in Fig. 6 with labeled parameters as labeling delay, classification delay, categorizing delay and overall response delay.

**Table 2:** Paradigm on categorization and validation matrix

**Demand Ration Validation**				
	**Device Density**	**Spectrum Demand**	**Categorized Devices**	**Occupancy rate**
**Server Driven**	88.32	73.21	32.11	64.24
**IoT Driven**	68.96	56.32	48.91	65.72
**IoMT Driven**	78.92	69.64	65.43	74.32
**AIoMT Driven**	82.12	75.43	73.28	80.62
**Time Ration Validation**				
	**Waiting Delay (ms)**	**Minimal Waiting Delay (ms)**	**Average Waiting Delay (ms)**	**Response Time (ms)**
**Server Driven**	9.34	4.32	3.11	2.42
**IoT Driven**	7.43	5.32	4.19	3.24
**IoMT Driven**	6.33	4.78	3.82	3.29
**AIoMT Driven**	4.31	4.93	4.21	2.97

**Table 3:** Representation of categorization parameters

	**Categorization Parameters (ms)**			**Overall response delay (ms)**
	**Labeling delay**	**Classification Delay**	**Categorizing delay**	
**IoT Devices**	12.23	8.34	56.53	18.42
**IoMT Devices**	11.32	10.43	69.32	24.32
**IoMT Labeled Devices**	10.43	9.31	53.32	22.43
**AIoMT General Devices**	8.432	7.423	32.32	22.02
**AIoMT Labeled Devices**	3.43	4.32	22.31	19.34

## Conclusion

This paper has discussed a novel approach for categorizing health monitoring devices under IoMT to Artificial Intelligence of Medical Things (AIoMT). The proposed technique is based on federated learning on acquiring information section through AIoMT devices coordination and attribute mapping with respect to block-alignment and coordination matrix collection. The technique has selected a local computation and aggregative server to extract initial categorization labels from primary layer of federated learning. The technique has successfully validated and resolved the conflict of multiple device resonance in the IoMT ecosystem. The proposed technique has retrieved a categorization label $\left( {\Delta {\mathbb{R}}} \right)$ with a higher order of device type classification and categorization. In near future the technique can be appended on sub-medical devices classification and categorization.

## Data Availability

The data that support the findings of this study will be available from the corresponding author upon request.
